# A Lumped Mass Model for Circular Micro-Resonators in Coriolis Vibratory Gyroscopes

**DOI:** 10.3390/mi10060378

**Published:** 2019-06-06

**Authors:** Xukai Ding, Jia JIA, Zhengcheng Qin, Zhihu Ruan, Liye Zhao, Hongsheng Li

**Affiliations:** 1 School of Instrument Science and Engineering, Southeast University, Nanjing 210096, China; 230169207@seu.edu.cn (J.J.); 230189281@seu.edu.cn (Z.Q.); 230179760@seu.edu.cn (Z.R.); liyezhao@seu.edu.cn (L.Z.); hsli@seu.edu.cn (H.L.); 2 Key Laboratory of Micro-Inertial Instruments and Advanced Navigation Technology, Ministry of Education, Nanjing 210096, China

**Keywords:** circular micro-resonators, coriolis vibratory gyroscopes, modal effective mass, modal equivalent force, electrostatic stiffness tuning

## Abstract

Coriolis vibratory gyroscopes (CVGs) with circular micro-resonators, such as hemispherical, ring, and disk resonators, exhibit excellent performances and have extraordinary potential. This paper discusses a generalized lumped mass model for both 3D and planar circular micro-resonators, establishing the relationship between the modal effective mass, the modal equivalent force, and the point displacement of the resonator. The point displacement description of a continuous circular resonator’s motion is defined from the view of capacitance measurement. The modal effective mass is, consequently, determined by the kinetic and the potential energy of the structure and is computed with numerical simulations. Moreover, the modal equivalent force, which can be theoretically calculated for any configuration of discrete electrodes, is deduced by using the concept of force density and the force distribution function. By utilizing the lumped mass model in this paper, the stiffness softening, the mode tuning, and the quadrature correction of the micro-resonators are investigated in detail. The theoretical model is verified by both the finite element method (FEM) and the experiments.

## 1. Introduction

MEMS Coriolis vibratory gyroscopes (CVGs) with circular micro-resonators, such as ring [1,2,3,4], disk [5,6], hemispherical [7,8], birdbath shell [9,10], and wineglass [11], exhibit extraordinary performances by their structural symmetry and, consequently, have drawn tremendous interest in both the academic and the industrial fields. The most significant benefit of the CVGs with circular micro-resonators is the ability of angular rate-integrating under the whole-angle operation [12,13,14], which has advantages in direct angle output, wide measurement range, fast response, and extensive dynamic range over the force re-balance operation of CVGs. Additionally, the high symmetry of the micro-resonator structure provides the possibility of in-situ calibration and compensation method, such as the well-known mode reversal [15,16,17], dual-mode actuation and sensing scheme [18,19], and multiple angular rate estimates [20,21]. These advantages attract researchers worldwide to CVGs with circular resonators.

Circular micro-resonators can be classified into two categories: 3D resonators, including micro ring and disk resonators, and planar resonators, such as hemispherical and birdbath shell resonators. 3D micro-resonators exhibit the considerable potential of performances but suffer significant fabrication challenges. Most of the research in this field focuses on optimizing the fabrication process. The fabrication process of planar micro-resonators is compatible with the widely served MEMS technology [22,23,24]. Researchers in this field aim their attention at optimizing structure designs to boost the Q factor of micro-resonators and to increase the mechanical sensitivity of CVGs [25,26].

To optimize structural designs and electrical control architectures, it is primarily to evaluate the dynamics of micro-resonators, in which modal effective mass and modal equivalent force play crucial roles. The pioneering work of [27] provides the method to evaluate the micro-resonator’s effective mass participating in motions. The physical parameters of 3D axis-symmetric gyroscopes, including effective mass, angular gain, and centrifugal mass, were derived in terms of the modal shape functions, and the procedures for numerically calculating the physical parameters are presented in ANSYS software. A procedure to identify mass and stiffness imperfections of micro ring gyroscopes is investigated in [28] in terms of parameter perturbations. The expressions of electrostatic force and electrostatic tuning were obtained by determining the derivatives of the electrical energy stored in capacitors.

However, currently, little research effort has been devoted explicitly to models addressing the relationships between the displacements, the effective mass, and the electrostatic force. There has been plentiful literature establishing continuous models for general MEMS devices [29,30,31,32,33,34] and, especially, for micro-gyroscopes [35,36,37]. Considering that the electrodes surround a circular resonator with a repetitive patten, we can establish a discrete model for circular micro-resonators in CVGs. This paper presents a general lumped mass model which discusses the modal effective mass and the modal equivalent force from the perspective of the point displacement description, clarifying the definitions of the point displacement and the modal effective mass, and examining the modal equivalent force with the concept of electrostatic force density in conjunction with force distribution functions.

The motivation of this paper is to establish the relationships between the displacements, the effective mass, and the electrostatic force and to quantitatively, readily investigate the effects of the stiffness softening and the electrostatic tuning of both 3D and planar micro-resonators, providing an efficient way to analyze the features of the mode matching and the quadrature correction. This paper is organized as follows. Section 2 gives the motion description of 3D or planar circular micro-resonators by a series of point displacements in view of capacitive sensing, leading to a general lumped mass model of a vibration system. Section 3 defines the modal effective mass of circular resonators from the perspective of point displacements, which are picked from capacitance measurements. The equivalent force exerted on the working modes is derived using the force density and the force distribution functions, establishing the complete lumped mass model for circular resonators in Section 4. As an application of the obtained model, the details of the stiffness softening, the mode matching, and the quadrature correction are thoroughly discussed in Section 5. Section 6 presents the experiments of the mode softening and the quadrature control of a micro ring resonator (MRR), verifying the theoretical analyses. Finally, Section 7 gives a brief conclusion of the paper.

## 2. Point Displacement Description of Motion

**Figure 1:**
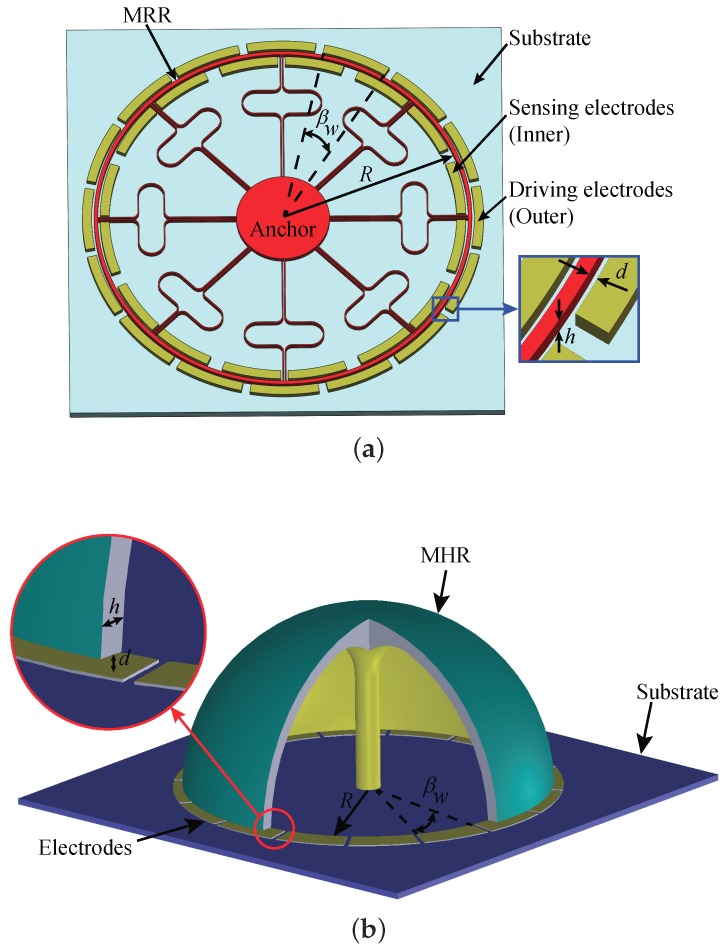
Circular micro-resonators in Coriolis vibratory gyroscopes (CVGs): (**a**) An micro ring resonator (MRR) with in-plane electrodes. (**b**) An micro hemispherical resonator (MHR) with in-plane electrodes.

**Figure 2:**
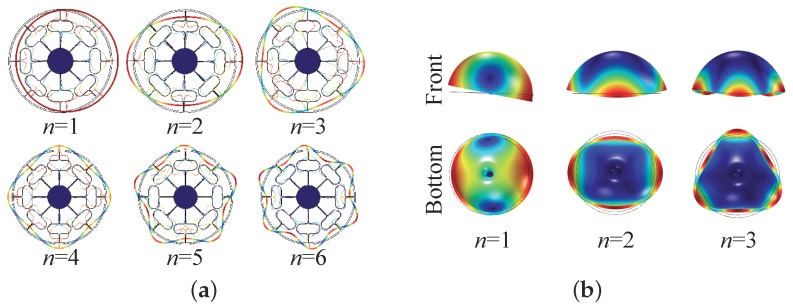
Flexural modes of the resonators: (**a**) First six in-plane flexural modes of an MRR. (**b**) First three flexural modes of an MHR.

Figure 1 shows an MRR and a micro hemispherical resonator (MHR) that can be utilized in CVGs. In Figure 1, *R* is the radius of the micro-resonator, *h* is the thickness of the structure, *d* is the static gap between the resonator and the actuation electrodes, as well as the sensing electrodes, and $\beta_{w}$ is the width of a single electrode. Figure 2 demonstrates the first few flexural mode shapes of the circular resonators depicted in Figure 1. The number *n* indicates the wave number along the circumference of the circular resonator. It should be noted that, for each *n*, there are two orthogonal mode shapes along their principal axes, although we only show one of the two in Figure 2. For CVGs operating in force re-balance operation, one of the two orthogonal modes is driven into stable resonance and, hence, is typically referred to as a driving mode. The other mode senses the applied angular rate via Coriolis coupling and is referred to as a sensing mode. For CVGs in whole-angle operation, the two orthogonal modes are identical and together provide the angle information of rotation. For convenience, in this paper, we refer to one of the two orthogonal modes as the cosine mode and the other as the sine one.

**Figure 3:**
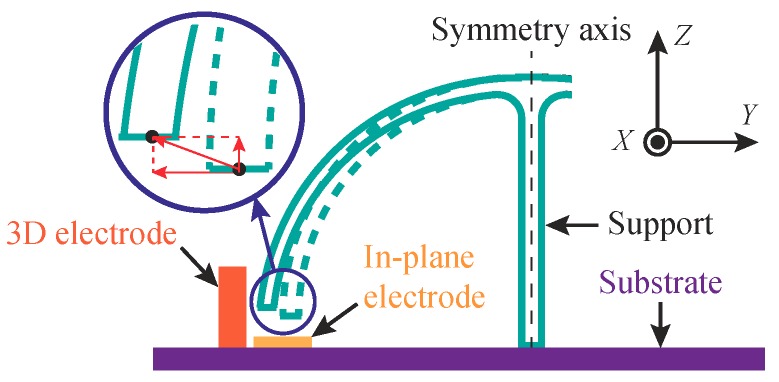
Implementations of the in-plane electrodes and the out-of-plane electrodes for the MHR.

The in-plane motion of an MRR is excited and sensed by in-plane electrodes surrounding the ring, as shown in Figure 1a. The mode shapes of an MHR have both out-of-plane components, revealed by front views in Figure 2b, and in-plane components, revealed by bottom views in Figure 2b. Either of the components can be utilized for sensing the motion of the micro-resonator, depending on the implementations of in-plane electrodes beneath the rim of the shell or 3D out-of-plane electrodes outside surrounding the shell, as illustrated in Figure 3. However, it is rather challenging to implement 3D electrodes with high aspect ratio in micro-fabrication processes. Figure 1b shows an in-plane electrode implementation, which is more compatible with the fabrication process. For a specific electrode implementation, only the motion that normal to the electrode surfaces, which constitute plate capacitors, can be effectively measured.

From the perspective of capacitive sensing, the displacement of a circular resonator can be generalized as

(1) $$r\left(\beta ,t\right)=\sum_{n=1}^{\infty }r_{nc}\left(t\right)\cos\left[n\left(\beta -\beta_{k,n}\right)\right]+\sum_{n=1}^{\infty }r_{ns}\left(t\right)\sin\left[n\left(\beta -\beta_{k,n}\right)\right],$$

where β∈(-π,π] is an axis angle along the circumference, $r_{nc}$ and $r_{ns}$ are point displacements along the principal axes of the cosine and the sine modes with the wave number of *n*, and $\beta_{k,n}$ is the azimuth of the corresponding principal axis. Displacements tangential to the electrodes and local vibrations, such as vibrations of supporting beams in a ring resonator, are not included in Equation (1).

In an ideal hemispherical shell, the angle of the principal axis is arbitrary for the perfect symmetry between the cosine and the sine modes. For an ideal ring resonator, such symmetry does not hold for some modes because of the discrete arrangement of supporting beams. As an example, in an MRR with eight sets of beams, the cosine modes with orders of n=4,8,12... are not symmetric to the sine ones, which can be readily verified in Figure 2a. For cases in which the modes with n=2 are the working modes, requirements of the suspension arrangements to obtain symmetric cosine and sine modes are discussed in [38]. In practice, micro-resonators always suffer structure asymmetry due to processing errors, making the modal principal axis towards a specific direction, $\beta_{k,n}$.

**Figure 4:**
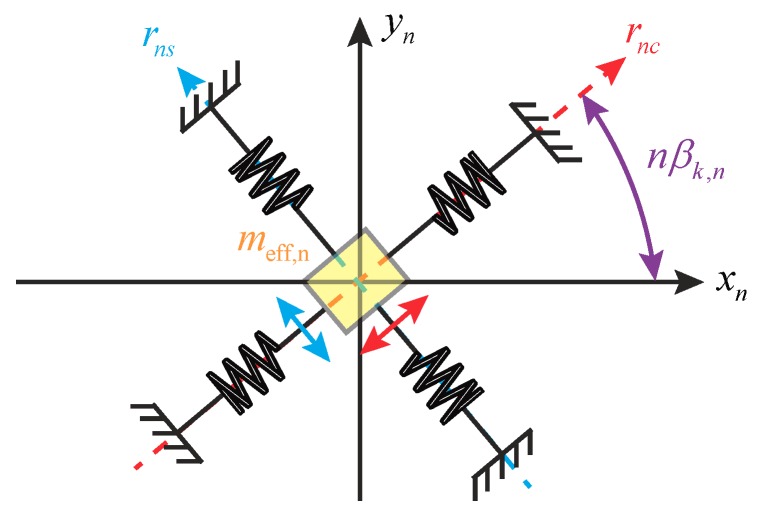
The lumped mass model of a circular resonator in CVGs.

Equation (1) reveals that the motion of a circular, continuous resonator can be represented in terms of a series of point displacements, $r_{nc}$ and $r_{ns}$, along its principal axes of each mode. By this method, it is possible to describe the resonator’s modal dynamics by a lumped mass model shown in Figure 4, where $x_{n}$ and $y_{n}$ are displacements of the resonator along $0^{\circ }$ and ${\left(0+90/n\right)}^{\circ }$ directions, respectively. In the next section, we will discuss the modal effective mass, $m_{\mathrm{eff},n}$, in Figure 4 from the perspective of point displacements.

## 3. Modal Effective Mass

The modal effective mass in the lumped mass model is directly related to the point displacements which describe the motion of the resonator. The above statement can be intuitively explained below. If the micro-resonator is excited by a force, the motion and the energy of the resonator are both determined. However, the point displacements which represent the resonator’s motion would scale according to the position of the measurement point. Therefore, if different point positions are chosen, the modal effective mass should also scale a factor to maintain consistent kinetic and potential energy.

The 3D displacement of a micro-resonator harmonically oscillating in the *n*-th mode can be expressed as [39]

(2) $$r\left(x\right)=r_{p}r_{n}\left(x\right),$$

where x is a position vector, $r_{p}$ is the effective displacement of the point at which the measurement is made, and $r_{n}\left(x\right)$ is a normalized mode shape of the *n*-th mode.

**Figure 5:**
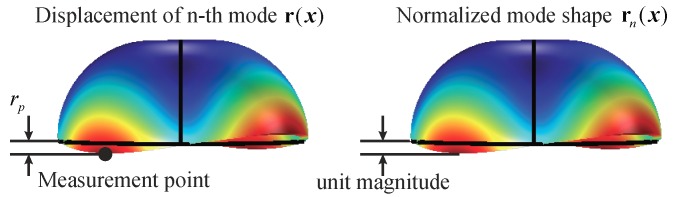
The normalization of an MHR with in-plane electrodes.

Figure 5 gives an example of the normalization of an MHR with in-plane electrodes. As mentioned in Section 2, in capacitive sensing, only the motion perpendicular to the electrodes can be effectively measured. Accordingly, in this example, the effective displacement $r_{p}$ is the vertical component at the measurement point, which is at the rim of the shell and along the resonator’s principal axis. The normalized mode shape is obtained such that the magnitude of the normalized mode shape’s deformation along $r_{p}$ is one. The normalization of an MRR, although not shown here, is by a similar approach.

In a harmonic oscillation with the wave number of *n*, the potential energy of a small volume dV at the position of x can be expressed as

(3) $$\begin{array}{cc} dE_{p} & =\frac{1}{2}\omega_{0,n}^{2}\;dm{\left|r(x)\right|}^{2}\\ & =\frac{1}{2}\omega_{0,n}^{2}\;\rho \left(x\right){\left|r_{p}r_{n}\left(x\right)\right|}^{2}\;dV, \end{array}$$

where $\omega_{0,n}$ is the *n*-th modal frequency, dm is the mass of the small volume, ρ(x) is the density of the resonator at the position of x.

The total potential energy of the micro-resonator can be evaluated by

(4) $$\begin{array}{cc} E_{p} & =\frac{1}{2}\omega_{0,n}^{2}{\left|r_{p}\right|}^{2}{\;\int \int \int \;}_{V}\rho \left(x\right){\left|r_{n}\left(x\right)\right|}^{2}dV\\ & =\frac{1}{2}\omega_{0,n}^{2}\;m_{\mathrm{eff},n}{\left|r_{p}\right|}^{2}, \end{array}$$

where

(5) $$m_{\mathrm{eff},n}≜{\;\int \int \int \;}_{V}\rho \left(x\right){\left|r_{n}\left(x\right)\right|}^{2}\;dV.$$

Equation (5) defines the modal effective mass. A simple solid mechanical finite element method (FEM) simulation can readily provide the modal frequencies and the corresponding effective masses. Table 1 presents the geometry parameters of the MRR shown in Figure 1a. Table 2 summarizes the first six modal frequencies and the effective masses of the ring, which are acquired via COMSOL Multiphysics. The material is set as single-crystal silicon with a density of 2330 kg/m${}^{3}$, Young’s modulus of 170 GPa, and Poisson’s ratio of 0.28.

Table 3 and Table 4 respectively give the structural parameters, and the modal frequencies and the effective masses of the MHR depicted in Figure 1b. The material in the FEM simulation is Pyrex glass with a density of 2230 kg/m${}^{3}$, Young’s modulus of 73 GPa, and Poisson’s ratio of 0.17.

It should be noted that, if the measurement point is chosen at another position which decreases the measurable displacement, the modal effective mass will increase due to the increment in $r_{n}\left(x\right)$ according to Equation (2). This increment in the modal effective mass does not suggest that more mass in the structure will participate in the harmonic oscillation. Instead, this increment comes from the reduction in the measured displacement, $r_{p}$, and, consequently, results in degradation of signal-noise-ratio.

Once the relationship between the point displacement and the modal effective mass is established, the lumped mass model for the *n*-th mode of a circular micro-resonator can be described as

(6) $$\varepsilon_{n}{\ddot{L}}_{n}+\frac{2}{\tau_{0,n}}\sigma_{n}{\dot{L}}_{n}+\omega_{0,n}^{2}\;\mu_{n}L_{n}=\frac{1}{m_{\mathrm{eff},n}}F_{n},$$

where

$$L_{n}={\left[\begin{array}{cc} x_{n} & y_{n} \end{array}\right]}^{T},\;F_{n}={\left[\begin{array}{cc} f_{x,n} & f_{y,n} \end{array}\right]}^{T},$$

$$\varepsilon_{n}=\left[\begin{array}{cc} 1+\varepsilon_{1,n} & \varepsilon_{2,n}\\ \varepsilon_{2,n} & 1-\varepsilon_{1,n} \end{array}\right],\;\sigma_{n}=\left[\begin{array}{cc} 1+\sigma_{1,n} & \sigma_{2,n}\\ \sigma_{2,n} & 1-\sigma_{1,n} \end{array}\right],\;\mu_{n}=\left[\begin{array}{cc} 1+\mu_{1,n} & \mu_{2,n}\\ \mu_{2,n} & 1-\mu_{1,n} \end{array}\right],$$

$\varepsilon_{n}$, $\sigma_{n}$, and $\mu_{n}$ are the mass, the damping coefficient, and the stiffness perturbation matrices; $\varepsilon_{1,n}$, $\varepsilon_{2,n}$, $\sigma_{1,n}$, $\sigma_{2,n}$, $\mu_{1,n}$, $\mu_{2,n}$ are unitless perturbations; $\tau_{0,n}$ is the decay time constant corresponding to the natural frequency of $\omega_{0,n}$; $f_{x,n}$ and $f_{y,n}$ are equivalent forces exerted on the *n*-th mode of the resonator, which will be investigated in Section 4.

In this paper, we assume the modes with n=2 are the working modes of CVGs and, thus, for convenience, the default subscript *n* will be omitted if n=2 modes are discussed in the context.

## 4. Modal Equivalent Force

### 4.1. Configurations of Actuation Electrodes for Micro Circular Resonators

To evaluate the modal equivalent forces in Equation (6), we necessarily discuss the configurations of actuation electrodes for MRRs and MHRs first.

**Figure 6:**
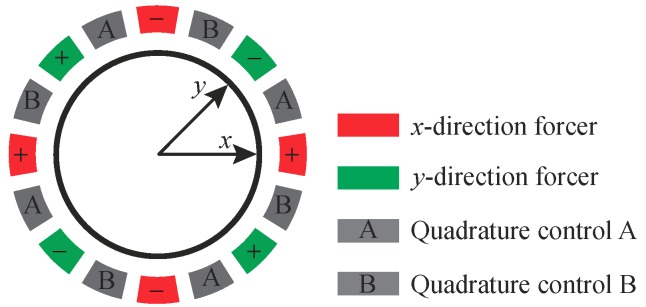
Electrode configuration for MRRs with 16 actuation electrodes.

In Figure 1a, the inner electrodes are used for motion pickoff, and the outer ones are used for actuation. Figure 6 gives a symmetric and differential configuration for MRRs with 16 actuation electrodes. Four electrodes are adopted for both *x*- and *y*- direction excitation, and eight for quadrature control. The “+”sign and the “−” sign in Figure 6 indicate the in-phase and the anti-phase relations of driving signals for mode shapes of n=2.

**Figure 7:**
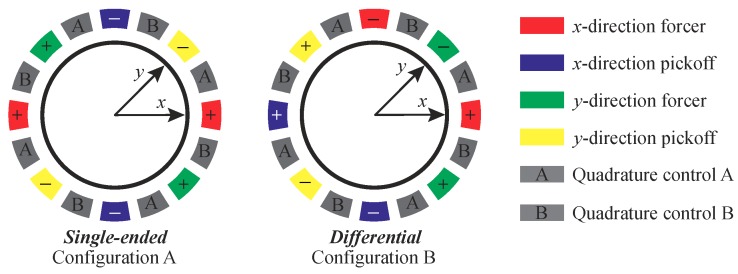
Two electrode configurations for MHRs with 12 actuation electrodes in total.

Different from MRRs, it is challenging to fabricate internal electrodes for MHRs. In Figure 1b, the MHRs are surrounded by 16 electrodes in total, which is a widespread practice. Generally, two of the electrodes are used for *x*-direction actuation, two for *x*-direction pickoff, two for *y*-direction actuation, two for *y*-direction pickoff, and eight for quadrature control. Figure 7 presents two possible configurations for MHRs. In Configuration A, the actuation and the pickoff electrodes are both symmetric but single-ended, whereas, in Configuration B, the arrangement is differential but asymmetric. In signal conditioning, differential pickoff has better common-mode rejection of interferences over single-ended arrangements. Many readout circuits inherently have differential architectures for capacitance pickoff [40,41,42,43,44]. The configuration of actuation electrodes for a wineglass resonator in [45] is similar to Configuration B.

### 4.2. Electrostatic Force Density

A single actuation electrode located at $\beta_{e}$ applied with an AC voltage of $\pm v_{a}\cos\left(\omega_{d}t\right)$ biased by a DC voltage of $v_{b}$ yields an electrostatic force density, in N/rad, between the grounded resonator and the actuation electrode as

(7) $$\begin{array}{cc} F_{D}\left(\beta ,t\right) & =\frac{\epsilon Rh}{2{\left[d-r\left(\beta ,t\right)\right]}^{2}}{\left[v_{b}\pm v_{a}\cos\left(\omega_{d}t\right)\right]}^{2}\\ & =\frac{\epsilon Rh}{2{\left[d-r\left(\beta ,t\right)\right]}^{2}}\left[v_{b}^{2}+\frac{1}{2}v_{a}^{2}\pm 2v_{b}v_{a}\cos\left(\omega_{d}t\right)+\frac{1}{2}v_{a}^{2}\cos\left(2\omega_{d}t\right)\right], \end{array}$$

where $\beta_{e}-\beta_{w}/2<\beta <\beta_{e}+\beta_{w}/2$, $\epsilon =8.85\times 10^{-12}\;F/m$ is the permittivity, $\omega_{d}$ is the angular frequency of the AC excitation voltage, and *R*, *h*, and *d* are as those defined in Figure 1.

Equation (7) reveals that the force contains three components: the static force, the fundamental force, and the second harmonic force. The static force will introduce electrostatic stiffness, which will be discussed in Section 5. The fundamental force is the excitation force, whereas the second harmonic force is an interference. If the Q value of the resonator is sufficiently high, the response to the force component with frequency of $2\omega_{d}$ can be ignored. Thus, the force density in Equation (7) can be approximated as

(8) $$F_{D}\left(\beta ,t\right)\approx \frac{\epsilon Rh}{2{\left[d-r\left(\beta ,t\right)\right]}^{2}}\left[\left(v_{b}^{2}+\frac{1}{2}v_{a}^{2}\right)\pm 2v_{b}v_{a}\cos\left(\omega_{d}t\right)\right].$$

By extending the force density from a single electrode to the entire actuation electrodes surrounding the structure, the overall force density along the resonator’s circumference can be written as

(9) $$\begin{array}{cc} F_{D}\left(\beta ,t\right)=\frac{\epsilon Rh}{2{\left[d-r\left(\beta ,t\right)\right]}^{2}} & [V_{x}^{2}S_{0x}\left(\beta \right)+2v_{bx}v_{ax}\cos\left(\omega_{d}t\right)S_{1x}\left(\beta \right)\\ & +V_{y}^{2}S_{0y}\left(\beta \right)+2v_{by}v_{ay}\cos\left(\omega_{d}t+\varphi \right)S_{1y}\left(\beta \right)+v_{A}^{2}S_{0A}\left(\beta \right)+v_{B}^{2}S_{0B}\left(\beta \right)], \end{array}$$

where

$$\begin{array}{c} V_{x}^{2}=v_{bx}^{2}+\frac{1}{2}v_{ax}^{2},\\ V_{y}^{2}=v_{by}^{2}+\frac{1}{2}v_{ay}^{2}, \end{array}$$

and $S_{0x}\left(\beta \right)$, $S_{0y}\left(\beta \right)$, $S_{0A}$, and $S_{0B}$ represent force distribution functions for the DC components of the *x*-, *y*-directional force, and the quadrature control A and B force, respectively; $S_{1x}\left(\beta \right)$ and $S_{1y}\left(\beta \right)$ for the fundamental components.

**Figure 8:**
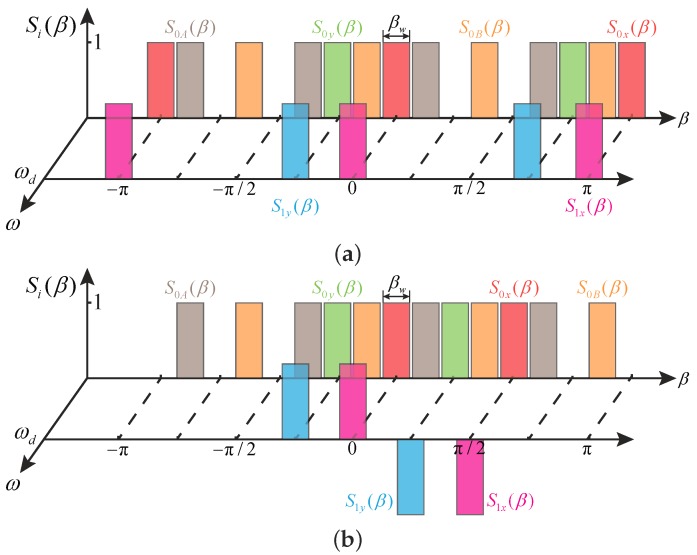
Force distribution functions of MHRs. (**a**) $S_{i}\left(\beta \right)$ for the single-ended electrode arrangement. (**b**) $S_{i}\left(\beta \right)$ for the differential electrode arrangement.

The force distribution function describes the angular position where the electrostatic force exists. As an example, Figure 8 demonstrates the force distribution functions of single-ended and differential electrode arrangements for MHRs. Each vertical bar in Figure 8 has an amplitude of one with a width of $\beta_{w}$, locating at the position where the corresponding electrode stands. The sign of each bar can be individually determined by the sign of each force component.

Due to the periodic feature, as illustrated in Figure 8, the force distribution functions can also be expressed in forms of Fourier series

(10) $$S_{i}\left(\beta \right)=\frac{1}{2}D_{i,0}+\sum_{n=1}^{\infty }D_{i,nc}\cos\left(n\beta \right)+\sum_{n=1}^{\infty }D_{i,ns}\sin\left(n\beta \right),\;\left(i=0x,0y,0A,0B,1x,1y\right)$$

where $D_{i,0}$, $D_{i,nc}$, and $D_{i,ns}$ are the coefficients of the series. Table 5 and Table 6 list the first four orders of the coefficients of $S_{i}\left(\beta \right)$ for the single-ended and the differential actuation electrodes of MHRs, respectively.

The terms in the tables suggest the effective coefficients of the electrostatic force exerted on the corresponding modes. If the coefficient is zero, the corresponding modes will not be excited. By comparing Table 5 and Table 6, it can be found that the single-ended and the differential arrangements have the same excitation efficiency for the working modes but will excite different interfering modes: the differential configuration will introduce n=1, n=3, and n=4 motions whereas the single-ended configuration will introduce n=4 motions.

Except for the motions of the working modes (n=2), all other motions are interferences, whose magnitude is determined by the Q value of the resonator and the frequency split between the interfering mode and the driving AC voltage. To prevent notable excitation of the interfering modes, the Q value and the frequency split should be sufficiently high and wide. The presence of the interfering motions comes from the excitation of a continuous structure by spatially discrete actuation electrodes.

The force distribution functions of MRRs, corresponding to Figure 6, can also be readily obtained via the above approach, and the Fourier coefficients of the distribution functions are listed in Table 7.

### 4.3. Equivalent Force on the Working Modes

By substituting Equation (10) into Equation (9), we can obtain an analytic expression for the electrostatic force density along the circumference of the resonator. Then, the equivalent force exerted on the *n*-th mode can be calculated by integrating the force density over the mode shape

(11) $$\left[\begin{array}{c} f_{x,n}\\ f_{y,n} \end{array}\right]=\int_{-\pi }^{\pi }F_{D}\left(\beta ,t\right)\left[\begin{array}{c} \cos\left(n\beta \right)\\ \sin\left(n\beta \right) \end{array}\right]d\beta .$$

The equivalent force in Equation (11) corresponds to the force in the lumped mass model described by Equation (6).

If the working modes of the resonator are effectively excited and the responses of the interfering modes are negligible, the displacement of the resonator can be expressed as

(12) $$r\left(\beta ,t\right)\approx x\left(t\right)\cos2(\beta -\beta_{k})+y\left(t\right)\sin2(\beta -\beta_{k}).$$

Assume that only the excitation voltages along the *x*-direction are applied, Equation (9) reduces to

(13) $$\begin{array}{cc} F_{D}\left(\beta ,t\right) & =\frac{1}{2}\epsilon Rh\left[\frac{1}{d^{2}}+\frac{2}{d^{3}}r\left(\beta ,t\right)+O\left(r^{2}\right)\right]\left[V_{x}^{2}S_{0x}\left(\beta \right)+2v_{bx}v_{ax}\cos\left(\omega_{d}t\right)S_{1x}\left(\beta \right)\right]\\ & \approx \frac{1}{2}\epsilon Rh\left\{\frac{1}{d^{2}}+\frac{2}{d^{3}}\left[x\left(t\right)\cos2(\beta -\beta_{k})+y\left(t\right)\sin2(\beta -\beta_{k})\right]\right\}[V_{x}^{2}S_{0x}\left(\beta \right)\\ & \;+2v_{bx}v_{ax}\cos\left(\omega_{d}t\right)S_{1x}\left(\beta \right)], \end{array}$$

where $O(r^{2})$ is infinitesimal of the displacement with orders higher than two and is neglected here for simplicity by considering that the displacements are relatively small compared to *d*. Associating Equations (10), (11) and (13) yields

(14) $$\begin{array}{cc} f_{x}\left(t\right) & =\frac{\epsilon Rh\pi }{2d^{2}}\left[D_{0x,2c}V_{x}^{2}+D_{1x,2c}\;2v_{bx}v_{ax}\cos\omega_{d}t\right]\\ & \;+\frac{\epsilon Rh\pi }{2d^{3}}\left[\left(D_{0x,0}+D_{0x,4c}\right)V_{x}^{2}+\left(D_{1x,0}+D_{1x,4c}\right)2v_{bx}v_{ax}\cos\omega_{d}t\right]x\\ & \;+\frac{\epsilon Rh\pi }{2d^{3}}\left[D_{0x,4s}V_{x}^{2}+D_{1x,4s}\;2v_{bx}v_{ax}\cos\omega_{d}t\right]y. \end{array}$$

By ignoring the static force and the high-frequency components in the electrostatic stiffness terms, Equation (14) can be further simplified as

(15) $$f_{x}\left(t\right)=\frac{\epsilon Rh\pi }{2d^{2}}\left(D_{1x,2c}\;2v_{bx}v_{ax}\cos\omega_{d}t\right)+\frac{\epsilon Rh\pi }{2d^{3}}\left(D_{0x,0}+D_{0x,4c}\right)V_{x}^{2}\;x+\frac{\epsilon Rh\pi }{2d^{3}}D_{0x,4s}V_{x}^{2}\;y.$$

Through this approach, if the *x*- and *y*-directional voltages and the quadrature control voltages are all applied, the equivalent electrostatic force exerted along the *x*-direction is

(16) $$\begin{array}{cc} f_{x}\left(t\right) & =\frac{\epsilon Rh\pi }{2d^{2}}\left[D_{1x,2c}\;2v_{bx}v_{ax}\cos\omega_{d}t+D_{1y,2c}\;2v_{by}v_{ay}\cos\left(\omega_{d}t+\phi \right)\right]\\ & \;+\frac{\epsilon Rh\pi }{2d^{3}}[\left(D_{0x,0}+D_{0x,4c}\right)V_{x}^{2}+\left(D_{0y,0}+D_{0y,4c}\right)V_{y}^{2}\\ & \;+\left(D_{0A,0}+D_{0A,4c}\right)v_{A}^{2}+\left(D_{0B,0}+D_{0B,4c}\right)v_{B}^{2}]x\\ & \;+\frac{\epsilon Rh\pi }{2d^{3}}\left[D_{0x,4s}V_{x}^{2}+D_{0y,4s}V_{y}^{2}+D_{0A,4s}v_{A}^{2}+D_{0B,4s}v_{B}^{2}\right]y, \end{array}$$

where ϕ is a phase difference between the *x*- and *y*-directional AC excitation voltages; $v_{A}$ and $v_{B}$ are DC voltages on the quadrature control A and B electrodes, respectively.

Similarly, the equivalent force along the *y*-direction is

(17) $$\begin{array}{cc} f_{y}\left(t\right) & =\frac{\epsilon Rh\pi }{2d^{2}}\left[D_{1x,2s}\;2v_{bx}v_{ax}\cos\omega_{d}t+D_{1y,2s}\;2v_{by}v_{ay}\cos\left(\omega_{d}t+\phi \right)\right]\\ & \;+\frac{\epsilon Rh\pi }{2d^{3}}\left[D_{0x,4s}V_{x}^{2}+D_{0y,4s}V_{y}^{2}+D_{0A,4s}v_{A}^{2}+D_{0B,4s}v_{B}^{2}\right]x\\ & \;+\frac{\epsilon Rh\pi }{2d^{3}}[\left(D_{0x,0}-D_{0x,4c}\right)V_{x}^{2}+\left(D_{0y,0}-D_{0y,4c}\right)V_{y}^{2}\\ & \;+\left(D_{0A,0}-D_{0A,4c}\right)v_{A}^{2}+\left(D_{0B,0}-D_{0B,4c}\right)v_{B}^{2}]y. \end{array}$$

Substituting the Fourier coefficients of the force distribution functions for MHRs into Equations (16) and (17) yields

(18) $$F=f+\left(K_{T}+K_{Q}\right)L,$$

where

$$\begin{array}{c} f=\frac{\epsilon Rh}{2d^{2}}\left[\begin{array}{c} 2\sin\beta_{w}\;2v_{bx}v_{ax}\cos\left(\omega_{d}t\right)\\ -2\sin\beta_{w}\;2v_{by}v_{ay}\cos\left(\omega_{d}t+\phi \right) \end{array}\right],\\ K_{T}=\frac{\epsilon Rh}{2d^{3}}\left[\begin{array}{cc} \left(2\beta_{w}+\sin2\beta_{w}\right)V_{x}^{2}+\left(2\beta_{w}-\sin2\beta_{w}\right)V_{y}^{2} & 0\\ 0 & \left(2\beta_{w}-\sin2\beta_{w}\right)V_{x}^{2}+\left(2\beta_{w}+\sin2\beta_{w}\right)V_{y}^{2} \end{array}\right],\\ K_{Q}=\frac{\epsilon Rh}{2d^{3}}\left[\begin{array}{cc} 4\beta_{w}\left(v_{A}^{2}+v_{B}^{2}\right) & 2\sin2\beta_{w}\left(v_{A}^{2}-v_{B}^{2}\right)\\ 2\sin2\beta_{w}\left(v_{A}^{2}-v_{B}^{2}\right) & 4\beta_{w}\left(v_{A}^{2}+v_{B}^{2}\right) \end{array}\right]. \end{array}$$

In Equation (18), $K_{T}$ represents the electrostatic stiffness introduced by the excitation voltages and $K_{Q}$ represents the electrostatic stiffness introduced by the quadrature control voltages. Equation (18) reveals that the excitation voltages will introduce the electrostatic stiffness along the excitation directions, whereas the quadrature control voltages will introduce both the stiffness along the excitation directions and the coupling stiffness across the directions. The impacts of $K_{T}$ and $K_{Q}$ on the working modes of the resonators will be discussed in Section 5.

Besides, it can be readily verified that the equivalent force exerted on the MRRs with the electrode configuration in Figure 6 is

(19) $$F=2f+\left(2K_{T}+K_{Q}\right)L.$$

## 5. Stiffness Softening and Electrostatic Stiffness Tuning

### 5.1. Stiffness Softening of the Working Modes

By substituting Equation (19) into Equation (6), we can get the complete form of the lumped mass model for the working modes of MRRs in CVGs

(20) $$\varepsilon \ddot{L}+\frac{2}{\tau_{0}}\sigma \dot{L}+\left[\omega_{0}^{2}\mu -\frac{1}{m_{\mathrm{eff}}}\left(2K_{T}+K_{Q}\right)\right]L=\frac{1}{m_{\mathrm{eff}}}2f.$$

**Figure 9:**
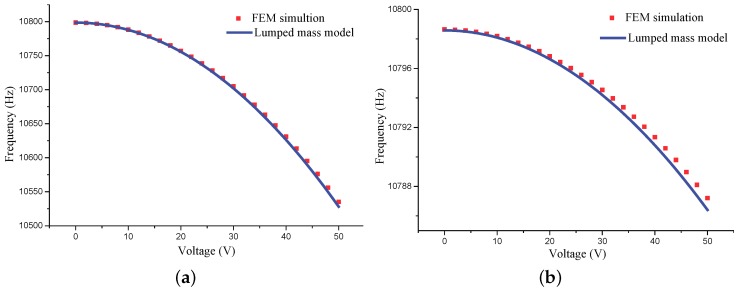
Modal frequency softening of the working modes of the MRR. (**a**) Frequency of the cosine mode. (**b**) Frequency of the sine mode.

Equation (20) indicates the electrostatic forces will introduce negative stiffness which softens the modal frequencies. Figure 9 presents the softening effect of the *x*-directional voltage on the modal frequencies of an ideal MRR. The FEM simulation was conducted by *Electromechanics* module of COMSOL. The results of the lumped mass model and the FEM simulation coincide very well within the low voltage range, whereas, in the high voltage range, the results demonstrate a minor discrepancy, which is believed from the approximation in Equation (13).

Figure 9 shows that the *x*-directional voltage significantly affects the modal frequency of the cosine mode and just slightly changes that of the sine mode. This phenomenon, together with the effect of the quadrature control voltages, can be utilized for correction of stiffness errors of the micro-resonators.

### 5.2. Electrostatic Stiffness Tuning

#### 5.2.1. Mode Matching

The condition of mode matching of the working modes is generally preferred in gyroscopic operations due to the high mechanical sensitivity. However, the non-ideal perturbations presenting in Equation (6) will undermine the symmetry of the micro-resonators and, accordingly, will lead to the frequency split of the working modes.

Considering the model described by Equation (20), the frequency matrix under the electrostatic force can be evaluated by

(21) $$\begin{array}{cc} {\omega_{E}}^{2} & =\omega_{0}^{2}\;\varepsilon^{-1}\mu -\frac{1}{m_{\mathrm{eff}}}\varepsilon^{-1}\left(2K_{T}+K_{Q}\right)\\ & \approx \omega_{0}^{2}{\left[\begin{array}{cc} 1+\varepsilon_{1} & \varepsilon_{2}\\ \varepsilon_{2} & 1-\varepsilon_{1} \end{array}\right]}^{-1}\left[\begin{array}{cc} 1+\mu_{1}-\kappa_{11} & \mu_{2}-\kappa_{12}\\ \mu_{2}-\kappa_{21} & 1-\mu_{1}-\kappa_{22} \end{array}\right], \end{array}$$

where

$$\begin{array}{c} \kappa_{11}=\frac{2\epsilon Rh\beta_{w}}{m_{\mathrm{eff}}\omega_{0}^{2}d^{3}}\left(v_{A}^{2}+v_{B}^{2}+2V_{x}^{2}\right),\\ \kappa_{12}=\kappa_{21}=\frac{2\epsilon Rh\beta_{w}}{m_{\mathrm{eff}}\omega_{0}^{2}d^{3}}\left(v_{A}^{2}-v_{B}^{2}\right),\\ \kappa_{22}=\frac{2\epsilon Rh\beta_{w}}{m_{\mathrm{eff}}\omega_{0}^{2}d^{3}}\left(v_{A}^{2}+v_{B}^{2}+2V_{y}^{2}\right), \end{array}$$

and the approximation is taken under $\sin\left(2\beta_{w}\right)\approx 2\beta_{w}$.

The modal frequencies of the resonator can be determined by the eigenvalues of ${\omega_{E}}^{2}$. Assume that the eigenvalues of ${\omega_{E}}^{2}$ are

(22) $$\begin{array}{c} {\tilde{\omega }}_{1}^{2}≜\lambda_{1}\omega_{0}^{2},\\ {\tilde{\omega }}_{2}^{2}≜\lambda_{2}\omega_{0}^{2}. \end{array}$$

where $\lambda_{2}\geq \lambda_{1}$. Then, we can obtain

(23) $$\lambda_{2}-\lambda_{1}=\frac{1}{1-\varepsilon_{1}^{2}-\varepsilon_{2}^{2}}\sqrt{M},$$

where

$$\begin{array}{c} M={\left[2-\kappa_{11}-\kappa_{22}-\varepsilon_{1}\left(\mu_{1}-\kappa_{11}\right)-\varepsilon_{1}\left(\mu_{1}+\kappa_{22}\right)-2\varepsilon_{2}\left(\mu_{2}-\kappa_{12}\right)\right]}^{2}\\ -4\left[1-\kappa_{11}-\kappa_{22}-\left(\mu_{1}-\kappa_{11}\right)\left(\mu_{1}+\kappa_{22}\right)-{\left(\mu_{2}-\kappa_{12}\right)}^{2}\right]\left(1-\varepsilon_{1}^{2}-\varepsilon_{2}^{2}\right). \end{array}$$

Solving

(24) $$\frac{\partial M}{\partial \kappa_{11}}=0,\;\frac{\partial M}{\partial \kappa_{12}}=0,\;\frac{\partial M}{\partial \kappa_{22}}=0,$$

gives the condition of $\lambda_{1}=\lambda_{2}$, namely the condition of mode matching,

(25) $$\begin{array}{c} \kappa_{11}=\frac{1}{\varepsilon_{2}}\left[\left(\kappa_{12}-\mu_{2}\right)\left(1+\varepsilon_{1}\right)+\varepsilon_{2}\left(1+\mu_{1}\right)\right], \end{array}$$

(26) $$\begin{array}{c} \kappa_{22}=\frac{1}{\varepsilon_{2}}\left[\left(\kappa_{12}-\mu_{2}\right)\left(1-\varepsilon_{1}\right)+\varepsilon_{2}\left(1-\mu_{1}\right)\right], \end{array}$$

(27) $$\begin{array}{c} \lambda_{1,2}=\frac{\mu_{2}-\kappa_{12}}{\varepsilon_{2}}. \end{array}$$

Equations (25)–(27) give the voltage requirements for mode matching

(28) $$\begin{array}{c} \left(1-\varepsilon_{1}\right)V_{x}^{2}-\left(1+\varepsilon_{1}\right)V_{y}^{2}=\frac{m_{\mathrm{eff}}\omega_{0}^{2}d^{3}}{2\epsilon Rh\beta_{w}}\left(\mu_{1}-\varepsilon_{1}\right)+\varepsilon_{1}\left(v_{A}^{2}+v_{B}^{2}\right),\\ \left(1-\varepsilon_{2}\right)v_{A}^{2}-\left(1+\varepsilon_{2}\right)v_{B}^{2}=\frac{m_{\mathrm{eff}}\omega_{0}^{2}d^{3}}{2\epsilon Rh\beta_{w}}\left(\mu_{2}-\varepsilon_{2}\right)+\varepsilon_{2}\left(V_{x}^{2}+V_{y}^{2}\right). \end{array}$$

Equation (28) indicates that the voltages to achieve matched modes are not unique. Mathematically, as long as the four voltage variables, $V_{x}$, $V_{y}$, $v_{A}$, and $v_{B}$, satisfy Equation (28), the modal frequencies of the MRR’s working modes will be identical. However, in CVGs the electrostatically tuned modal frequencies are desired to be as close to $\omega_{0}$ as possible, which implies that the least electrostatic effort accomplishes the mode matching.

By combining Equations (25)–(27), we can have the tuned frequency offset ratio

(29) $$\begin{array}{cc} \frac{\omega_{0}^{2}-{\tilde{\omega }}_{1,2}^{2}}{\omega_{0}^{2}} & =1-\lambda_{1,2}\\ & =\frac{2\epsilon Rh\beta_{w}}{m_{\mathrm{eff}}\omega_{0}^{2}d^{3}}\left(V_{x}^{2}+V_{y}^{2}+v_{A}^{2}+v_{B}^{2}\right). \end{array}$$

**Figure 10:**
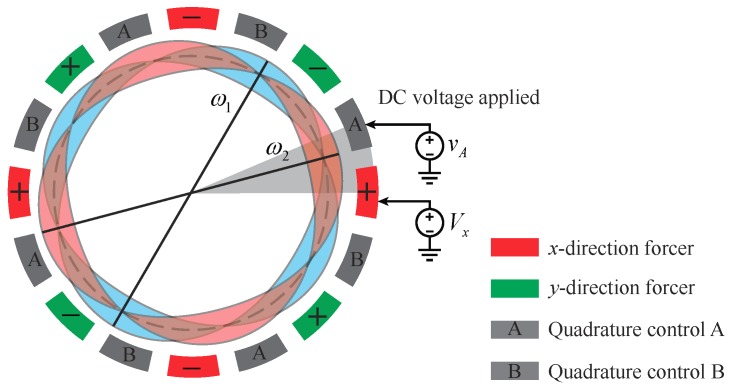
Configuration of the tuning electrodes for non-ideal micro-resonators.

Equation (29) suggests that, to obtain the minimum value of the frequency offset, $V_{x}$ or $V_{y}$ and $v_{A}$ or $v_{B}$ can be set to zero according to signs of $\mu_{1}-\varepsilon_{1}$ and $\mu_{2}-\varepsilon_{2}$. For example, if $\mu_{1}>\varepsilon_{1}$, then $V_{y}$ can be set as zero; and if $\mu_{2}>\varepsilon_{2}$, then $v_{B}$ can be set to zero. This procedure can be illustrated by Figure 10, where the principal axes of the modal frequencies are denoted as $\omega_{1}$ and $\omega_{2}$ ($\omega_{2}>\omega_{1}$). If the azimuth of $\omega_{2}$ is between the *x*-directional electrodes and the quadrature control A electrodes, these two sets of electrodes should be applied with proper voltages to achieve the mode-matched situation.

In the above example, the voltages to achieve the mode matching are

(30) $$\begin{array}{c} V_{x}^{2}=\frac{m_{\mathrm{eff}}\;\omega_{0}^{2}d^{3}}{2\epsilon Rh\beta_{w}}\frac{\mu_{1}-\varepsilon_{1}+\varepsilon_{1}\mu_{2}-\varepsilon_{2}\mu_{1}}{1-\varepsilon_{1}-\varepsilon_{2}}, \end{array}$$

(31) $$\begin{array}{c} v_{A}^{2}=\frac{m_{\mathrm{eff}}\;\omega_{0}^{2}d^{3}}{2\epsilon Rh\beta_{w}}\frac{\mu_{2}-\varepsilon_{2}-\varepsilon_{1}\mu_{2}+\varepsilon_{2}\mu_{1}}{1-\varepsilon_{1}-\varepsilon_{2}}. \end{array}$$

In addition, the minimum value of Equation (29) with the boundary condition of Equation (28) can be found as

(32) $$1-\lambda_{1,2}=\frac{\left|\mu_{1}-\varepsilon_{1}\right|+\left|\mu_{2}-\varepsilon_{2}\right|}{1-\varepsilon_{1}-\varepsilon_{2}},$$

which reveals that, under the electrostatic stiffness tuning, the closest frequency to $\omega_{0}$ is

(33) $${\tilde{\omega }}_{1,2}=\omega_{0}\sqrt{1-\frac{\left|\mu_{1}-\varepsilon_{1}\right|+\left|\mu_{2}-\varepsilon_{2}\right|}{1-\varepsilon_{1}-\varepsilon_{2}}}.$$

**Figure 11:**
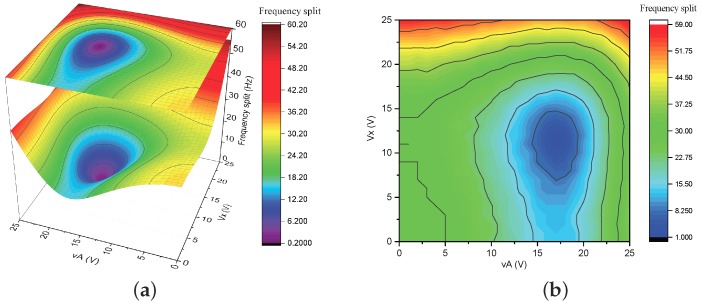
Frequency split of the non-ideal MRR under control of different $V_{x}$ and $v_{A}$. (**a**) Analytical result. (**b**) Finite element method (FEM) simulation.

Figure 11a, which is directly from the eigenvalues of Equation (21), demonstrates the frequency split of a non-ideal MRR under the control of different $V_{x}$ and $v_{A}$. The perturbations satisfy that $\mu_{1}-\varepsilon_{1}=1.48$‰ and $\mu_{2}-\varepsilon_{2}=2.57$‰, leading to an initial frequency split of 32Hz and the azimuth of $\omega_{2}$ as $15^{\circ }$. In Figure 11a, the voltage requirement of mode matching is $V_{x}=11.96\;V$ and $v_{A}=15.75\;V$, which is consistent with Equations (30) and (31). Figure 11b gives the result of the FEM simulation of the electrostatic tuning procedure. The non-ideal resonator in the simulation was obtained by adding mass perturbation according to the form of $50\cos4(\beta -15^{\circ })\;{\mathrm{kg}/m}^{3}$, providing an initial frequency split of 32Hz and the azimuth of $\omega_{2}$ as $15^{\circ }$. The FEM simulation confirms the analytical result presented in Figure 11a and, thus, verifies the electrostatic stiffness features predicted by the model of Equation (20).

#### 5.2.2. Quadrature Correction

The anti-diagonal terms in the frequency matrix, ${\omega_{E}}^{2}$, will deviate the azimuth of the principal axes from the directions of the excitation and pickoff electrodes and will result in coupling vibration between the *x*- and *y*-directions. This coupling vibration is quadrature to the motion induced by the Coriolis effect and is generally referred to as the quadrature signal.

By ignoring the second order infinitesimals in Equation (21), ${\omega_{E}}^{2}$ can be reduced to

(34) $${\omega_{E}}^{2}\approx \omega_{0}^{2}\left[\begin{array}{cc} 1+\mu_{1}-\varepsilon_{1}-\kappa_{11} & \mu_{2}-\varepsilon_{2}-\kappa_{12}\\ \mu_{2}-\varepsilon_{2}-\kappa_{12} & 1-\mu_{1}+\varepsilon_{1}-\kappa_{22} \end{array}\right],$$

which suggests that $\kappa_{12}$ alone can be used to eliminate the anti-diagonal terms, indicating that the voltage difference applied on quadrature control electrodes can almost electrostatically correct the quadrature coupling and, consequently, align the principal axes of the working modes towards the electrodes.

**Figure 12:**
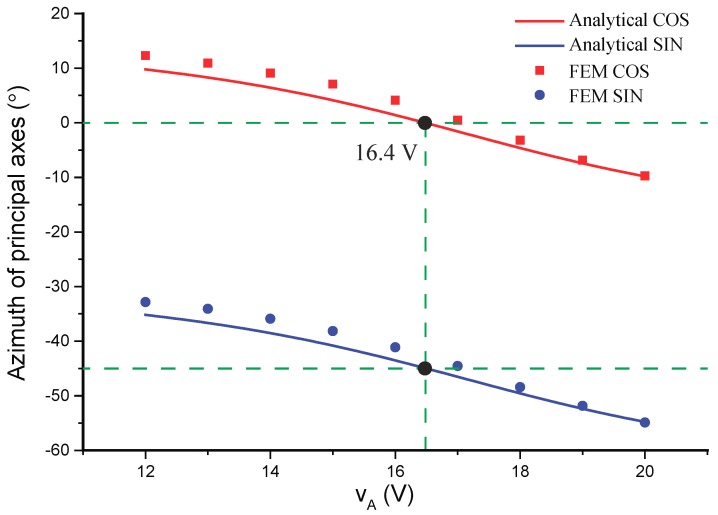
Analytical and FEM simulations of the azimuth of the principal axes under the control of $v_{A}$.

Figure 12, demonstrating the shift of the principal axes under the control of different $v_{A}$, validates the capability of the quadrature control electrodes to suppress the coupling signal. The azimuth of the cosine mode gradually rotates to $0^{\circ }$ as the control voltage $v_{A}$ achieves 16.4V, whereas the axis of the sine mode is along $\pm 45^{\circ }$.

In addition, it should be noted that the working modes cannot achieve matched until the quadrature coupling is canceled, which means the quadrature correction is the precondition of the mode matching.

## 6. Experiments

**Figure 13:**
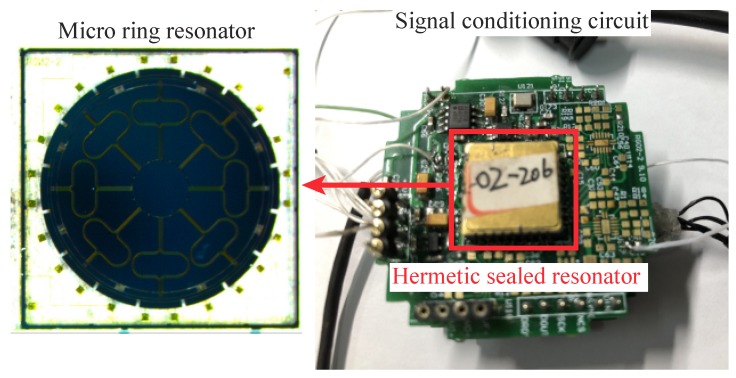
The ring resonator and the signal conditioning circuits.

Figure 13 demonstrates a silicon MRR along with the signal conditioning circuits. The design parameters of the MRR is listed as in Table 1. The theoretical modal effective mass is $3.488\times 10^{-7}\;\mathrm{kg}$ and the modal frequencies of the working modes of the device under test are 9489.9Hz and 9500.9Hz, which were extracted with $v_{bx}=v_{by}=5\;V$ and $v_{ax}=v_{ay}=0.75\;V$ by a signal generator (Agilent 33220A, Keysight, Santa Rosa, CA, USA) and a multimeter (Agilent 34401A, Keysight). The capacitance readout circuit is based on a ring-diode interface circuit discussed in [44].

**Figure 14:**
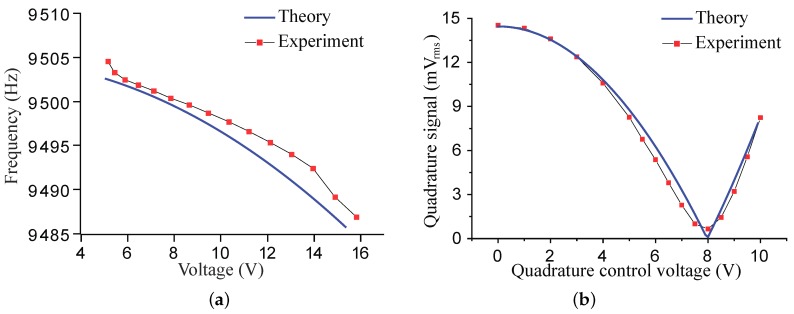
(**a**) The mode softening. (**b**) The relationships between the quadrature signal and the quadrature control voltage.

**Figure 15:**
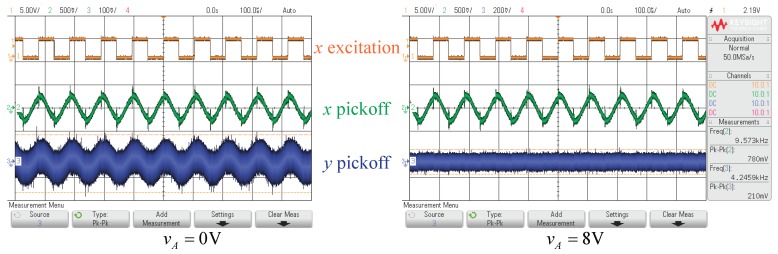
The quadrature signals before and after the quadrature correction.

Figure 14a gives the predicted and the experimental results of the modal frequency softening. The voltage applied to the electrodes along the *x*-direction was increased gradually and the modal frequencies of the working modes decreased as expected. Figure 14b presents the curves of the relations between the quadrature control voltage and the quadrature signal. The quadrature signal was recorded by the multimeter and was captured by an oscilliscope (Keysight InfiniiVision DSOX2024A) simultaneously. The quadrature coupling signal dropped to the minimum value greater than zero when the quadrature control voltage reached 8V, while the theoretical prediction suggests that the quadrature can achieve an exact zero. The slight deviation is from the background noise in the circuit, as displayed in Figure 15.

The consistency between the theoretical and the experimental characteristics of the electrostatic stiffness tuning and the quadrature control validates the modal effective mass and the equivalent force respectively discussed in Section 3 and Section 4.

## 7. Conclusions

This paper demonstrated a general lumped mass model for both 3D and planar circular micro-resonators in CVGs, specifically associating the modal effective mass and the modal equivalent force with the point displacement of the continuous circular structure. The point displacements from the capacitance measurement position were utilized to represent the motion of the structure and, then, the corresponding modal effective mass was obtained by keeping the kinetic and the potential energy constant at different measurement points. The modal effective mass of the micro-resonators was calculated by FEM software. Then, the modal equivalent force was derived by introducing the electrostatic force density and the force distribution function. By this approach, the stiffness softening and the electrostatic tuning were studies analytically, revealing the exact frequency change and the angle shift of the modal principal axes under specific voltages. The theoretical results were validated by both numerical simulations and experiments.

## Figures and Tables

**Table 1:** Parameters of the MRR.

Parameters	Values	Units
*R*	3000	μm
*h*	120	μm
*d*	5.2	μm
Ring width	80	μm
Beam width	20	μm
Anchor radius	750	μm
$\beta_{w}$	21	${}^{\circ }$

**Table 2:** Modal frequencies and corresponding effective masses of the MRR.

Mode	Frequency	Effective Mass	Mode	Frequency	Effective Mass
cos	(Hz)	($\times 10^{-7}$ kg)	sin	(Hz)	($\times 10^{-7}$ kg)
n=1	5344.3	5.336	n=1	5344.6	5.336
n=2	10,799	3.488	n=2	10,799	3.488
n=3	23,772	4.016	n=3	23,774	4.016
n=4	41,077	4.722	n=4	43,774	5.789
n=5	85,983	2.410	n=5	85,986	2.409
n=6	118,590	2.263	n=6	118,590	2.263

**Table 3:** Parameters of the MHR.

Parameters	Values	Units
*R*	3800	μm
*h*	200	μm
*d*	5	μm
$\beta_{w}$	21	${}^{\circ }$

**Table 4:** Modal frequencies and corresponding effective masses of the MHR.

Mode	Frequency	Effective Mass	Mode	Frequency	Effective Mass
cos	(Hz)	($\times 10^{-5}$ kg)	sin	(Hz)	($\times 10^{-5}$ kg)
n=1	4659.1	1.152	n=1	4659.2	1.152
n=2	16,529	1.936	n=2	16,529	1.936
n=3	44,699	2.370	n=3	44,700	2.370
n=4	81,745	2.835	n=4	81,752	2.834
n=5	125,517	3.338	n=5	125,521	3.338
n=6	174,952	3.914	n=6	174,961	3.916

**Table 5:** Fourier coefficients of $S_{i}\left(\beta \right)$ for the single-ended electrode arrangement of MHRs.

*n*		0	1	2	3	4
$S_{0x}\left(\beta \right)$	$D_{0x,nc}$	$2\beta_{w}/\pi$	0	$2\sin\left(\beta_{w}\right)/\pi$	0	$\sin\left(2\beta_{w}\right)/\pi$
	$D_{0x,ns}$		0	0	0	0
$S_{0y}\left(\beta \right)$	$D_{0y,nc}$	$2\beta_{w}/\pi$	0	0	0	$-\sin\left(2\beta_{w}\right)/\pi$
	$D_{0y,ns}$		0	$-2\sin\left(\beta_{w}\right)/\pi$	0	0
$S_{0A}\left(\beta \right)$	$D_{0A,nc}$	$4\beta_{w}/\pi$	0	0	0	0
	$D_{0A,ns}$		0	0	0	$2\sin\left(2\beta_{w}\right)/\pi$
$S_{0B}\left(\beta \right)$	$D_{0B,nc}$	$4\beta_{w}/\pi$	0	0	0	0
	$D_{0B,ns}$		0	0	0	$-2\sin\left(2\beta_{w}\right)/\pi$
$S_{1x}\left(\beta \right)$	$D_{1x,nc}$	$2\beta_{w}/\pi$	0	$2\sin\left(\beta_{w}\right)/\pi$	0	$\sin\left(2\beta_{w}\right)/\pi$
	$D_{1x,ns}$		0	0	0	0
$S_{1y}\left(\beta \right)$	$D_{1y,nc}$	$2\beta_{w}/\pi$	0	0	0	$-\sin\left(2\beta_{w}\right)/\pi$
	$D_{1y,ns}$		0	$-2\sin\left(\beta_{w}\right)/\pi$	0	0

**Table 6:** Fourier coefficients of $S_{i}\left(\beta \right)$ for the differential electrode arrangement of MHRs.

*n*		0	1	2	B	4
$S_{0x}\left(\beta \right)$	$D_{0x,nc}$	$\frac{2\beta_{w}}{\pi }$	$2\sin\left(\beta_{w}/2\right)/\pi$	0	$2\sin\left(3\beta_{w}/2\right)/3\pi$	$\sin\left(2\beta_{w}\right)/\pi$
	$D_{0x,ns}$		$2\sin\left(\beta_{w}/2\right)/\pi$	0	$-2\sin\left(3\beta_{w}/2\right)/3\pi$	0
$S_{0y}\left(\beta \right)$	$D_{0y,nc}$	$\frac{2\beta_{w}}{\pi }$	$2\sqrt{2}\sin\left(\beta_{w}/2\right)/\pi$	0	$-2\sqrt{2}\sin\left(3\beta_{w}/2\right)/3\pi$	$-\sin\left(2\beta_{w}\right)/\pi$
	$D_{0y,ns}$		0	0	0	0
$S_{0A}\left(\beta \right)$	$D_{0A,nc}$	$\frac{4\beta_{w}}{\pi }$	0	0	0	0
	$D_{0A,ns}$		0	0	0	$2\sin\left(2\beta_{w}\right)/\pi$
$S_{0B}\left(\beta \right)$	$D_{0B,nc}$	$\frac{4\beta_{w}}{\pi }$	0	0	0	0
	$D_{0B,ns}$		0	0	0	$-2\sin\left(2\beta_{w}\right)/\pi$
$S_{1x}\left(\beta \right)$	$D_{1x,nc}$	0	$2\sin\left(\beta_{w}/2\right)/\pi$	$2\sin\left(\beta_{w}\right)/\pi$	$2\sin\left(3\beta_{w}/2\right)/3\pi$	0
	$D_{1x,ns}$		$-2\sin\left(\beta_{w}/2\right)/\pi$	0	$2\sin\left(3\beta_{w}/2\right)/3\pi$	0
$S_{1y}\left(\beta \right)$	$D_{1y,nc}$	0	0	0	0	0
	$D_{1y,ns}$		$-2\sqrt{2}\sin\left(\beta_{w}/2\right)/\pi$	$-2\sin\left(\beta_{w}\right)/\pi$	$-2\sqrt{2}\sin\left(3\beta_{w}/2\right)/3\pi$	0

**Table 7:** Fourier coefficients of $S_{i}\left(\beta \right)$ for the electrode arrangement of MRRs.

*n*		0	1	2	B	4
$S_{0x}\left(\beta \right)$	$D_{0x,nc}$	$4\beta_{w}/\pi$	0	0	0	$2\sin\left(2\beta_{w}\right)/\pi$
	$D_{0x,ns}$		0	0	0	0
$S_{0y}\left(\beta \right)$	$D_{0y,nc}$	$4\beta_{w}/\pi$	0	0	0	$-2\sin\left(2\beta_{w}\right)/\pi$
	$D_{0y,ns}$		0	0	0	0
$S_{0A}\left(\beta \right)$	$D_{0A,nc}$	$4\beta_{w}/\pi$	0	0	0	0
	$D_{0A,ns}$		0	0	0	$2\sin\left(2\beta_{w}\right)/\pi$
$S_{0B}\left(\beta \right)$	$D_{0B,nc}$	$4\beta_{w}/\pi$	0	0	0	0
	$D_{0B,ns}$		0	0	0	$-2\sin\left(2\beta_{w}\right)/\pi$
$S_{1x}\left(\beta \right)$	$D_{1x,nc}$	0	0	$4\sin\left(\beta_{w}\right)/\pi$	0	0
	$D_{1x,ns}$		0	0	0	0
$S_{1y}\left(\beta \right)$	$D_{1y,nc}$	0	0	0	0	0
	$D_{1y,ns}$		0	$-4\sin\left(\beta_{w}\right)/\pi$	0	0

## Abbreviations

The following abbreviations are used in this manuscript:

CVG	Coriolis vibratory gyroscope
FEM	finite element method
MHR	micro hemispherical resonator
MRR	micro ring resonator
